# Supplementary material for: Constitutive turnover of histone H2A.Z at yeast promoters requires the preinitiation complex
Source: eLife. 2016 Jul 20;5:e14243. doi: 10.7554/eLife.14243 (PMC4995100; doi:10.7554/eLife.14243)
Supplement: Supplementary file 1. — DOI: http://dx.doi.org/10.7554/eLife.14243.046 [file elife-14243-supp1.docx]

**Supplemental File 1:** Table of yeast strains used in this study

| **Strain** | **Genotype** | **Source/reference** | **Method** |
| --- | --- | --- | --- |
| *W303* | *ade2-1 trp1-1 can1-100 leu2-3,112 his3-11,15 ura3 GAL psi^+^* | Standard Strain |  |
| *HHY221 (yEL044)* | *W303 MATa TOR1-1 fpr1∆::loxP-LEU2-loxP rpl13a::RPL13A-2×FKBP12-loxP* | (Haruki et al., 2008) |  |
| *HHY170*  *(yEL066)* | *W303 MAT*α *TOR1-1 fpr1∆::natMX4 rpl13a::RPL13A-2xFKBP12-TRP1 rpb1::RPB1-FRB::kanMX6* | (Haruki et al., 2008) |  |
| *HHY209*  *(yEL073)* | *W303 MAT*α *TOR1-1 fpr1∆::natMX4 rpl13a::RPL13A-2xFKBP12-TRP1 spt15::SPT15-FRB-GFP::kanMX6* | (Haruki et al., 2008) |  |
| *yEL098* | *W303 MATa TOR1-1 fpr1∆::loxP-LEU2-loxP rpl13a::RPL13A-2×FKBP12-loxP spt15::SPT15-FRB-GFP-kanMX6* | This study | Tetrad segregant of HHY221 and HHY209 |
| *yEL090* | *W303 MAT*a *TOR1-1 fpr1∆::natMX4 rpl13a::RPL13A-2xFKBP12-TRP1 rpb1::RPB1-FRB::kanMX6* | This study | Tetrad segregant of HHY221 and HHY170 |
| *yEL123* | *HHY221 INO80-FRB-GFP::HISMX6* | This study | PCR-based integration using pFA6a-*FRB-GFP-HIS3MX6* (EUROSCARF: P30581) |
| *yEL152* | *HHY221 htz1::HTZ1-2xFLAG-URA3* | This study | PCR-based integration using pRS416-HTZ1-2xFLAG (pEL353) as template |
| *yEL154* | *yEL098 htz1::HTZ1-2xFLAG-URA3* | This study | PCR-based integration using pRS416-HTZ1-2xFLAG (pEL353) as template |
| *yEL170* | *yEL090 htz1::HTZ1-2xFLAG-URA3* | This study | PCR-based integration using pRS416-HTZ1-2xFLAG (pEL353) as template |
| *yEL189* | *yEL123 htz1::HTZ1-2xFLAG-URA3* | This study | PCR-based integration using pRS416-HTZ1-2xFLAG (pEL353) as template |
| *yEL219* | *yEL152 SWC5-FRB::HISMX6* | This study | PCR-based integration using pFA6a-*FRB-HIS3MX6* (EUROSCARF: P30579) |
| *yEL220* | *yEL154 SWC5-FRB::HISMX6* | This study | PCR-based integration using pFA6a-*FRB-HIS3MX6* (EUROSCARF: P30579) |
| *yEL297* | *W303 MATa TOR1-1 fpr1∆::P RPL13A-2XFKBP12::P KIN28-FRB RBP3-3xFLAG-p-kanMX-p* | This study | PCR-based integration using p3xFLAG p-kanMX-p (pEL362) as template (Mizuguchi G. et al. 2004) |
