# Supplementary figures and images for: Constitutive turnover of histone H2A.Z at yeast promoters requires the preinitiation complex
Source: eLife. 2016 Jul 20;5:e14243. doi: 10.7554/eLife.14243 (PMC4995100; doi:10.7554/eLife.14243)

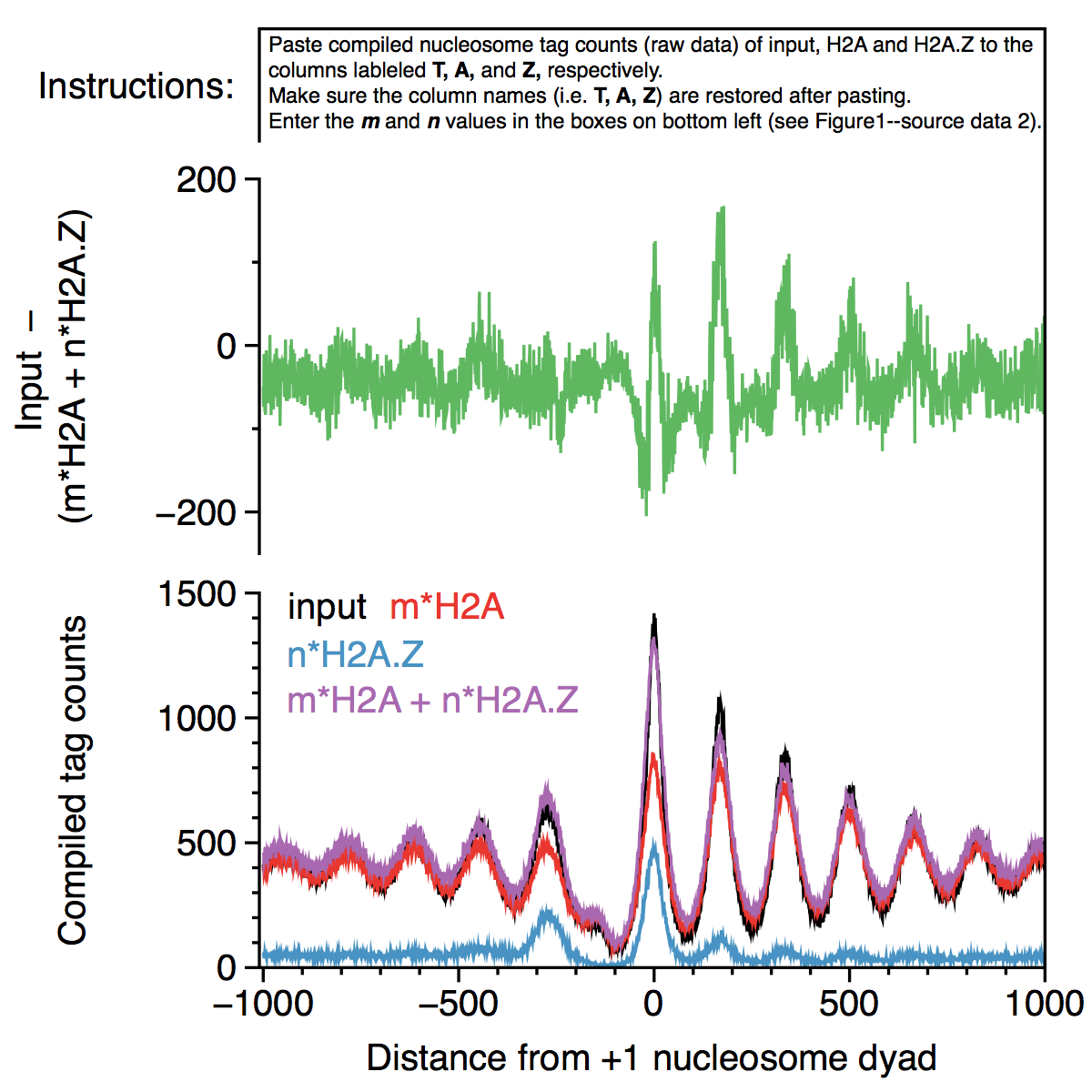

Supplement: Supplementary file 3. — DOI: http://dx.doi.org/10.7554/eLife.14243.048 [file elife-14243-supp3.zip › Supplemental_file_3_TAZ_Normalization_visualizer.dgraph/QuickLook/Preview.png]
