# Supplementary material for: Constitutive turnover of histone H2A.Z at yeast promoters requires the preinitiation complex
Source: eLife. 2016 Jul 20;5:e14243. doi: 10.7554/eLife.14243 (PMC4995100; doi:10.7554/eLife.14243)

Additional procedure for TAZ normalization

The TAZ_Normalization.py program (**Source code 1**) is a Python script used to calculate the normalization factors ***m*** and ***n*** for each IP reaction (technical replicate) in a qChIP-seq experiment. The "INFILE" should be a tab-delimited text file (UTF-8 Unix format) organized as follows:

```
position      input     H2A     H2A.Z
-1000         1173      937     644
-999          1198      930     597
-998          1213      847     604
:             :         :       :
:             :         :       :
:             :         :       :
998           1224      1137    380
999           1275      1077    385
1000          1263      1065    395
```

Column 1 indicates the position (in basepairs) relative to the +1 dyad. Columns 2, 3, and 4 indicate tag counts of the input, H2A (flow-through), and H2A.Z (IP) fractions, respectively. The top row is the header. Rows 2 to 2002 are consecutive data points from the -1000 to +1000 bp positions relative to the +1 dyads. Two example "toy" files are provided in **Supplemental file 5** and **file 6**. Each file represents a technical replicate (an individual IP reaction) from the *TBP-FRB* strain with or without rapamycin treatment, respectively. An example of how to execute the program at the Terminal on a Mac computer is shown below:

```
your_computer$ TAZ_normalization.py Supplemental_file_5.txt
```

To configure the computer to run the python script, please refer to Chapter 6 of *Practical Computing for Biologists* by Haddock S.H.D. and Dunn C.W.

Upon executing the program, two prompts will request the read counts in the "No-Z-zones" of the input fraction and the H2A fraction. These values will be used to calculate the ***m*** value for scaling the H2A profile. To scale the H2A.Z (IP) profile, the script will determine an ***n*** value between 0 and 1 such that ***R***, the TAZ curve fitting score, is minimum. ***R*** is calculated by the following equation:

$$\sum_{i=-500}^{501} \{[input_i - (\boldsymbol{m} \times H2A_i + \boldsymbol{n} \times H2A.Z_i)]^2 \times input_i{}^2\}$$

where *i* represents the incremental positions between -500 and +500 bp around the +1 dyad. An error message will appear if the local minimum is not found.

To visualize the result of the normalization procedure, download the "TAZ_Normalization_visualizer.dgraph" file, which can be found in **Supplemental file 3**. This file runs on Datagraph (*Visual Data Tools*), which can be downloaded here: http://visualdatatools.com/DataGraph/Download/index.html

In Datagraph, open the TAZ_Normalization_visualizer.dgraph file. Copy and paste the content of the toy file (e.g. TBP_FRB_no_RAP.txt) into the four columns labeled Position, T, A, and Z. Make sure the column names for T, A, and Z are restored after pasting. Then enter the calculated ***m*** and ***n*** values in the boxes on the bottom left.

A screen shot of the TAZ_Normalization_visualizer.dgraph is shown below. The complete datasets for the qChIP-seq experiments, including the technical and biological replicates, can be found in **Figure 1—source data 3-5**, **Figure 3—source data 3**, **Figure 4—source data 1** and **Figure 6—source data 1**. The normalization factors can be found in **Figure_1—source_data_2**,

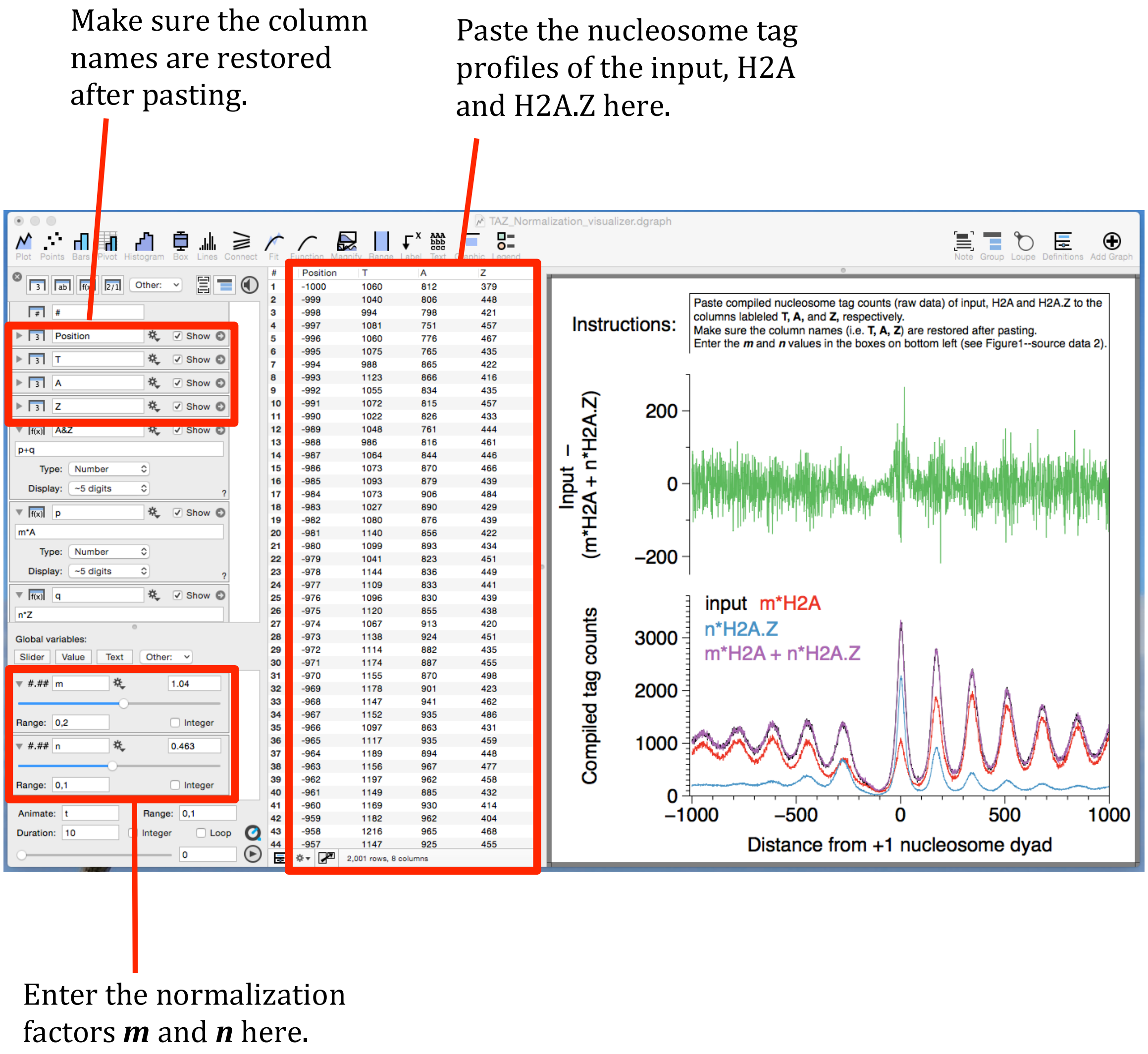

Supplement: Supplementary file 4. — DOI: http://dx.doi.org/10.7554/eLife.14243.049 [file elife-14243-supp4.pdf]
